# COVID-19 and the Use of Masks by Children. Statement From the Association of Schools of Public Health in the European Region and the European Academy of Paediatrics

**DOI:** 10.3389/fped.2021.580150

**Published:** 2021-01-28

**Authors:** Henrique Lopes, John Middleton, Ann De Guchtenaere, Adamos Hadjipanayis

**Affiliations:** ^1^Public Health Unit, Institute of Health Sciences, Catholic University of Portugal, Lisbon, Portugal; ^2^The Association of Schools of Public Health in the European Region (ASPHER), Brussels, Belgium; ^3^The European Academy of Paediatrics, Brussels, Belgium; ^4^Medical School, European University Cyprus, Nicosia, Cyprus

**Keywords:** COVID-19, masks, children, mask use, children safety

## Abstract

Despite the fact that the use of masks and respirators in adults has already reached a consensus in almost all countries and for situations in which they are recommended, this is not the case for the use of mask by children. This statement, regarding the usage of mask by children, has been jointly produced by the Association of Schools of Public Health in the European Region (ASPHER) and the European Academy of Paediatrics (EAP). It provides recommendations on the size of the mask, the material and ergonomics of children's masks. The authors also discuss the psychological dimension of children when they are asked to wear a mask. Moreover, they tackle the difficulties of children with disabilities.

## Introduction

The Association of Schools of Public Health in the European Region (ASPHER) is the key independent European organization dedicated to strengthening the role of public health by improving education and training of public health professionals (1). The European Academy of Paediatrics (EAP) exists to promote the health of children and young people in Europe.

The ASPHER follows the principle that all action in Public Health must be based on scientific evidence, as it is the only way to guarantee the best health care to populations.

Previously, another Statement was produced by ASPHER, dedicated to the use of masks and respirators in general (1). The mandatory use of masks and the particularities of its use by children led ASPHER and the EAP to develop this Statement (2).

The use of masks and respirators in adults has reached a consensus in almost all countries, which is not being verified for children. Therefore, this work aims at providing guidance on the use of masks by children, for each different age groups' specificities, as what has been produced is always directed to adults. A call of research is also intended due to the lack of knowledge and theorisation regarding almost everything in this subject.

For this article, a bibliographic survey was conducted about the use of masks by children, with very scarce literature being found. To fill in the knowledge gaps, it was considered important to strengthen recommendations based on experience. To make the best possible recommendations, interviews were conducted with nurses and medical doctors working in pediatric hospital settings. In October and November 2020, people from more than 20 countries were also consulted regarding the use of mask by children in the school environment, being also in discussion with over 20 other people in the ASPHER Senior Board, UNESCO and school health organizations. In all the situations, the lack of scientific production available on the subject was appointed, representing a clear need for further research.

## Recommendations by Aspher and EAP

**1. Masks can provide the same type of protection in the context of COVID-19 to a child as to an adult**. The use of masks should be considered without hesitation under the commonly adopted conditions, especially when in large gatherings and when social distancing is difficult to maintain (3, 4). It should be noted that, for different reasons, masks offer different levels of protection as rated by an IQR scale (5), with a lesser protection degree in children than in adults.
2. Although there is some manufacture of masks appropriately sized for children, their **availability is rare even in hospital facilities and almost impossible to acquire during the pandemic**. On the contrary, home-made masks or those produced by the clothing industry can address adequate sizes and adjust to supply demands.
  In the early pandemic, we received the information that only one mask size (for adults) was available in the hospital environment, unlike what happens with all other medical devices. This led to the need of adapting masks for children, having neither the consistent dimension nor the ergonomics required, the loss of effectiveness would be likely, along with increased discomfort, decreased adherence and use compliance. Most recent information state that due to the massification of the mask market, it is easier for children-sized masks to be available in hospitals. Nonetheless, child-sized masks must be made consistently available (6). Funding is also necessary for studies assessing mask adherence and efficacy in pediatric populations.
3. The material and ergonomics of children's masks must respect some basic principles:
  a. **Only masks with elastic bands should be used**. Masks that need to be laced are more difficult to use and adequately fit children.
  b. **Ergonomic design is also critical**. The function of a mask is achieved if air passes only through the fabric. Large masks allow air to pass through the sides, thus reducing its safety.
  c. **Design stamping is very important**. There is vast experience in pediatrics that children react better to decorated materials with cartoon drawings and images from the children's universe. This is also true with masks, as children react much better to social masks made with fabrics decorated with cartoon images than to typical surgical masks.
  d. As stated above, **masks that fit the size of children's heads** are lacking. This issue is particularly important due to different age groups having different head and shape dimensions.
  e. In the few studies that exist on the subject, **children mainly complain of the heat and humidity** induced by masks (7, 8).

4. Many manufacturers have come up with child hat-shield solutions (a hat with 360° plastic protections around the child's head, covering the shoulders). It is a very interesting solution, particularly for the age groups between 2 and 6 years old. **However, it should be noted that it is not an exact substitute for mask use**:
  a. The mask in the COVID-19 context essentially has a protective function regarding other individuals during the asymptomatic phase (9, 10).
  b. The visor hat-shield might protect the child from droplets, but as with adult face-shields, there is no scientific evidence that it protects other individuals.
  c. The degree of protection afforded to the direct entry of droplets is partly lost, as SARS-CoV-2 infected droplets can remain deposited for hours or days on the plastic. Being within reach of the child's hands, it is an immediate surface for handling.

5. **For children, masks represent a relevant psychological dimension**, which unlike for adults must be understood in a 2-fold approach (physical and psychological). It is important to consider the issues related both to the masks used by children and by adults with which they live. The recognition of family members and other close loved ones is largely based on facial traits. In very young children (under 4 years old) fear is often verified toward the person wearing a mask. Training is needed for people wearing masks who have close contact with children. For example, it is helpful to play with the child by successively putting on and taking off the mask, thus turning this learning into a child's play.
6. **As with adults, the adoption of a policy of mandatory use of masks by children must be accompanied by training in use and disposal** (11). Children tend to have more physical contact between peers than adults, similarly to contacts with surfaces, touching the face with less caution, etc. As a consequence, the risk of incorrect use of the masks can jeopardize the advantages of mask portability (12). Note that incorrect compliance in the mask use might not be due to a failure in the concept of wearing masks, but due to failure in the training of those responsible for providing masks to the child.
7. **Unless there is a specific medical recommendation, only masks should be considered for children**, especially if having pre-existent allergies or dermatitis. Respirators [FFP2/FFP3 (N95)] should not be used by children due to the following reasons:
  a. Respirators are less comfortable, which generates lesser compliance and children get tired if the use is for long periods (13).
  b. There are no respirators fit for children (14). As respirators are less plastic and adaptable, there is lesser efficiency when an ill-fitted size is used.
  c. The purpose of respirator use was designed and studied for adults and professionals, not for children (15).

8. **When considering the use of masks by children, distinctions should be made for at least four age groups:**
  a. 0–2 years old. No advantage was found in its use and despite no literature being yet published, recent recommendations against mask use by this age group where made by the Japan Pediatric Society and Centers for Disease Control and Prevention (CDC) and the American Academy of Pediatrics (AAP) (16, 17) due to possible risks.
    i. There may be an exception when children go to a hospital with COVID-19 patients or when they are exposed to higher contagion risk. Even in these cases, one must weigh between the potential gains and losses not only related to contagion, but also to the psychological difficulties that the child may have with comfort, etc. The decision to require a mask should only be taken by the Hospital Pediatric Service.
    ii. For prostrate children, mask portability is much easier, and their natural resistance will be reduced. This condition is always a clinical sign that there may be a need to reinforce the child's protection, and the decision of mask use/non-use should be defined by the assisting doctor.
    iii. It must be noted that the mask use in children of this age group includes the risk of the mask being removed and possible breathing difficulties due to the multiple layers of fabric/tissue, as noted in the CDC's recommendation for cloth face coverings (14).

b. 3–4 years old. The practical experience with this age group is that children are less resistant to wearing masks but are often afraid of being approached by adults who wear this equipment, with crying being frequent in these situations.
  i. Mask use should be recommended/imposed whenever children go to a hospital or other clinical setting.
  ii. Parents/guardians have a critical role in appeasing, deconstructing fear and training their children. The best approach to achieve this is by playing with a mask with children. Also, the design of the child's mask is very important for its acceptance. Particularly in this age group, it is essential to use masks made with elastics only.

c. 5–6 years old.
  i. The approach suggested above for 3–4-year-olds is identical but crying, and other manifestations of fear are much less frequent. For the rational explanation of the use of masks, one can begin by providing instructions for adequate compliance, non-manipulation, etc.

d. Above 6 years old.
  i. The portability of the mask is very similar to that of adults.
  ii. Communication about the use of mask, its placement, disposal, etc., must be adapted to the pedagogical needs of each age group, namely as to the instructions' form (more or less dependent on graphics) and depth. The differentiation of contents is suggested by the following age groups:
    1. 6–10 years old.
    2. 11–14 years old.
    3. Above 14 years old.

9. **The use and correction of the use of masks by children are directly linked to their parents' education** (18).
  a. The protection degree of each child is thus largely dependent on social inequalities, with differences of almost three times regarding the compliance of mask use.
  b. All children should have support from teachers, especially those in less educational-qualified families.
  c. Ideally, parents should also be trained by the School, establishing a School-Parental educational partnership.

10. As in adults, **the mask should not be considered to be a panacea nor a unique solution for COVID-19 protection**. As referred in the Statement on the use of masks in adults (1), this measure should always be included within the context of other Non-Pharmaceutical Measures (NPMs) and be taught as part of this broader hygienic system.
11. All children are unique. Thus, **the relation of disability to the imposition of wearing masks must be pondered in each case**. The effective protection provided by the correct use of a mask must be considered against the loss of cognitive, emotional and relational, among other dimensions of the child's life and family. It must be ensured that children have no loss of citizenship due to a limitation in health that poses a difficulty or hinders the use of masks, namely the freedom of movement on an equal basis with non-sick peers, nor that he may be subject to sanctions for surpassing the mandatory use of mask.
12. **Particular care should be taken when deciding to put masks on children who previously have a disability** (19). Three groups stand out:
  a. Those in which masks can limit the child's relation with the world. The most frequent case is that of deaf children where sign language is part of their basic communication system. The use of masks can limit or hinder this essential socialization process. In a balance between avoidance of contagion and loss of communication, the effective risk that the child incurs must be carefully weighed. The risk of contagion is probabilistic, and the risk of communication loss is a certainty.
  b. Children in which health problem promotes rapid mask degradation. Many syndromes promote continuous and abundant drooling, which leads to a change in the mask's permeability, a potential increase in respiratory effort, and great discomfort for the child. In these cases, it is recommended that there is no imposition of mask use. Otherwise, children who are in these circumstances can easily be limited in their citizenship, such as the use of public transport, entry into stores, and other spaces that require the use of masks.
  c. Those who by mental affectation do not support the use of masks. Perhaps the most frequent situation, but not the only, is that of the autistic children. Forcing the use of masks can jeopardize months or years of pedagogical support, social inclusion, loss of confidence in people who work on that child's autism, etc. It is also not acceptable for children to be penalized for this. A possible solution could be to use visors, if applicable. There may also be a negative reaction in these children toward professionals who work with them that do not have their faces visible. Likewise, the use of a visor may be a good option (20).

## Data Availability Statement

The original contributions presented in the study are included in the article/supplementary material, further inquiries can be directed to the corresponding author/s.

## Author Contributions

HL: main writer of the article. JM, AD, and AH: contributors for the article's writing and endorsers by corresponding international organizations. All authors contributed to the article and approved the submitted version.

## Conflict of Interest

The authors declare that the research was conducted in the absence of any commercial or financial relationships that could be construed as a potential conflict of interest.
